# Multiple Hypovascular Tumors in Kidney: A Rare Case Report and Differential Diagnosis

**DOI:** 10.1155/2013/595193

**Published:** 2013-12-03

**Authors:** Pei-Yu Wu, Sheng-Fung Lin, Ping-Hsun Wu, Yi-Chun Tsai, Yu-Ting Kuo, Mei-Chuan Kuo, Hung-Chun Chen

**Affiliations:** ^1^Division of Nephrology, Department of Internal Medicine, Kaohsiung Medical University Chung-Ho Memorial Hospital, No. 100 Tzyou 1st Road, Kaohsiung 80756, Taiwan; ^2^Division of Hematology and Oncology, Department of Internal Medicine, Kaohsiung Medical University Chung-Ho Memorial Hospital, Kaohsiung, Taiwan; ^3^Department of Medical Imaging, Kaohsiung Medical University Chung-Ho Memorial Hospital, Kaohsiung, Taiwan; ^4^Faculty of Renal Care, College of Medicine, Kaohsiung Medical University, Kaohsiung, Taiwan

## Abstract

The most common malignant renal tumor is renal cell carcinoma and surgery is the standard treatment. The proportion of lymphoma with renal involvement is 2~15% and lymphoma could be cured by chemotherapy without nephrectomy. Sonography, computed tomography (CT), and magnetic resonance imaging (MRI) can detect and characterize a renal mass. We present a case of right renal hypovascular tumors and differential diagnosis of hypovascular tumors by image study. CT scan showed hypovascular tumors and MRI image revealed multifocal hypovascular solid tumors with significantly increased apparent diffusion coefficient (ADC) of diffusion weighted imaging (DWI). Based on image finding, renal lymphoma was highly suspected. Renal lymphoma was confirmed by renal biopsy and this patient received chemotherapy without surgery. The noninvasive CT scan and MRI image can help clinicians to diagnose the characteristics of renal mass and to avoid unnecessary nephrectomy.

## 1. Introduction

The most common incidental renal malignant tumor is renal cell carcinoma and renal lymphoma is rare. Sonography, computed tomography (CT), and magnetic resonance imaging (MRI) can detect and characterize a renal mass. The accurate diagnosis of a renal mass depends on many factors, including the clinical history, physical examination, image study, and sometimes renal biopsy may be considered. Thus, we present a case of right renal hypovascular tumors, which was confirmed as renal lymphoma by renal biopsy. Further differential diagnosis of a hypovascular tumor from image study was also discussed in this paper.

## 2. Case Presentation

**Figure 1:**
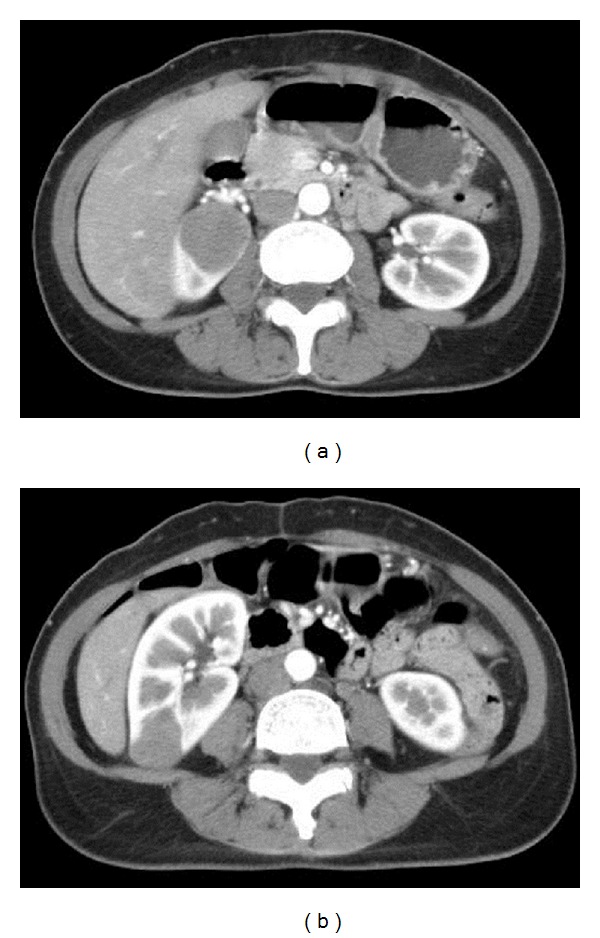
Abdominal computed tomography (CT) scan. Abdominal CT scan enhancement showed hypovascular tumors at upper poles (a), lower poles (b), and middle portions of the right kidney.

**Figure 2:**
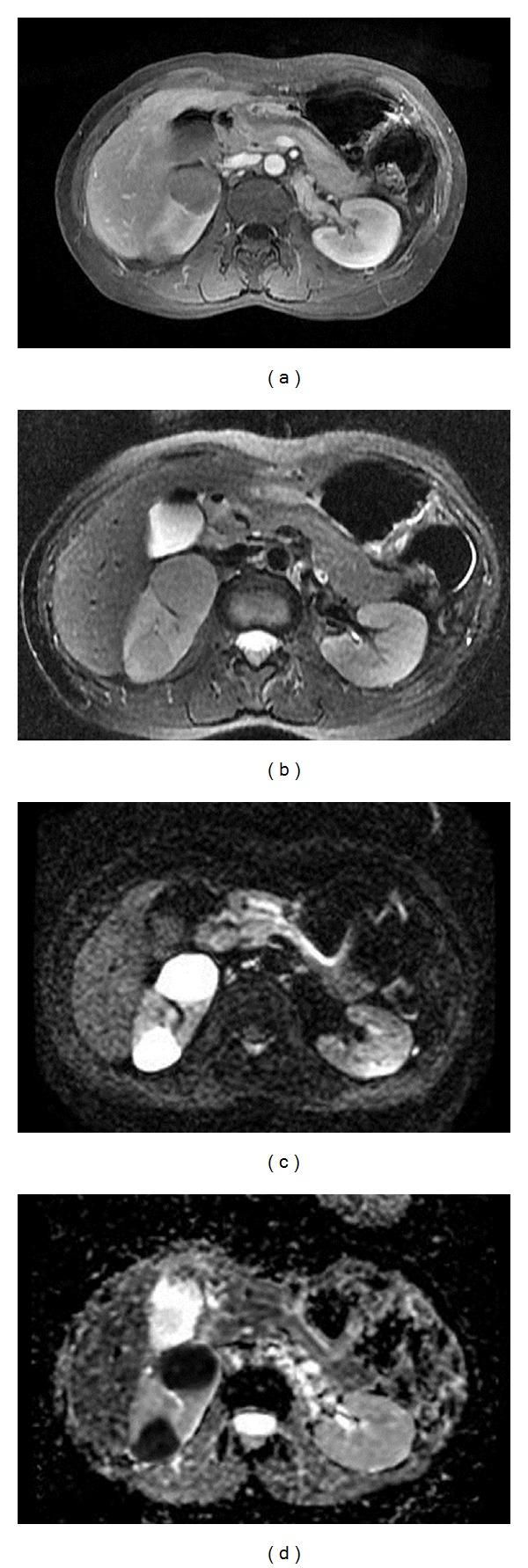
Abdominal magnetic resonance imaging (MRI). Abdominal MRI showed right kidney multiple lesions without enhancement during arterial phase of dynamic study (a), hypointense masses on T2-weighted images (b), and multifocal hypovascular hypercellular solid tumors of the right kidneys with bright lesion in diffusion weighted imaging (DWI) (c) and significantly decreased apparent diffusion coefficient (ADC) (d).

**Figure 3:**
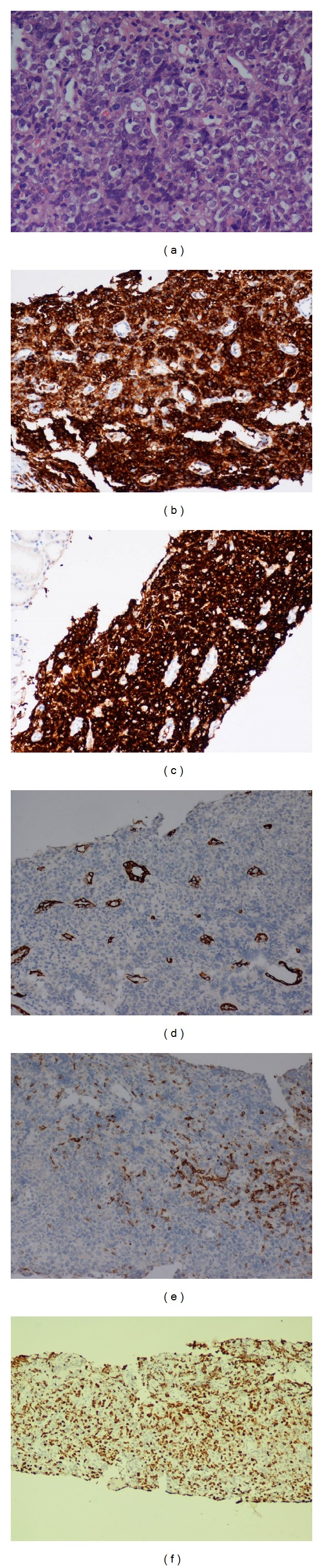
Renal pathology. Renal cortex replaced by medium-sized to large atypical lymphoid cells. The tumor cells show high nucleocytoplasmic ratio, prominent eosinophilic nucleoli, and single cell apoptosis in haemotoxylin eosin stain ×100 (a). The tumor cells are positive for LCA ×100 (b), CD20 ×100 (c), but negative for CK ×100 (d), CD3 ×100 (e), and Ki-67 stain ×100 (f) shows the positivity focally up to 60%.

A 52-year-old female had experienced intermittent epigastralgia with hunger pain and right flank pain without radiation for three months. Urine routine revealed microscopic hematuria with RBC count of 3–5 in high power field. Normocytic anemia with hemoglobin level of 11.3 g/dL without leukopenia or thrombocytopenia was shown. Neither abnormal coagulopathy nor liver and renal dysfunction were found. Abdominal sonography demonstrated a right hypo-echogenic renal mass at right upper and lower pole without hydronephrosis or kidney enlargement. Furthermore, abdominal CT showed multiple hypovascular tumors at the upper and lower pole and middle portions of the right kidney with sizes 3.3 cm, 2.5 cm, and 2.8 cm, respectively, without metastatic lymphadenopathy (Figure 1). Abdominal MRI revealed multifocal hypovascular solid tumors with significantly increased apparent diffusion coefficient (ADC) of diffusion weighted imaging (DWI) with enhancement during arterial phase of dynamic study and left renal hilar lymphadenopathy (Figure 2). Based on the imaging findings, CT-guide renal biopsy was performed. Renal pathology revealed renal cortex replaced by medium-sized to large atypical lymphoid cells and tumor cells, which are positive for Leucocyte Common Antigen (LCA), CD20, and CD10 immunohistochemical stain (Figure 3). Diffuse large B cell lymphoma was the confirmed diagnosis and Positron Emission tomography-computed tomography (PET/CT) showed the involvement in right kidney and stomach, compatible with diffuse large B cell lymphoma (stage IV). Normocellular bone marrow and normal chromosome were found by bone marrow biopsy. The patient received 6 cycles of cyclophosphamide, doxorubicin, vincristine, and prednisone plus rituximab chemotherapy. However, the patient expired because of severe infection.

## 3. Discussion

The most common malignant renal tumor is renal cell carcinoma (80~85%) and transitional cell carcinoma (approximately 8%). Traditionally, renal biopsy has been indicated for evaluation of significant proteinuria, hematuria, or associated renal disease. Renal biopsy is usually not suggested for diagnosis of renal cell carcinoma because of the risk of tumor seeding from biopsy tract [1]. On the other hand, renal involvement in lymphoma is noted about 2~15% cases in CT scan finding which shows hypovascular characteristics [2, 3]. Conversely, renal biopsy has been regarded as the diagnostic tool of hypovascular renal tumor to avoid unnecessary nephrectomy regardless of primary or secondary lymphoma [4]. Thus, differential diagnosis of renal tumor in image study is important, especially renal lymphoma. Renal lymphoma may represent as single or multiple sonolucent or weakly echogenic masses on ultrasound [5]. However, this finding is nonspecific and not enough to confirm the diagnosis. Typical CT patterns of renal lymphoma include single or multiple masses, invasion from contiguous retroperitoneal disease, perirenal disease, and diffuse renal infiltration [6, 7]. In enhanced CT scan, renal lymphoma usually represents nonenhancement hypovascular tumor [8]. On the contrary, most clear cell renal cell carcinoma usually shows the enhancement [9], and only 15.9% displays the hypovascular or avascular tumor [10]. It is difficult to differentiate lymphoma in CT imaging. In this case, enhanced CT scan showed hypovascular tumors, which is not the typical CT finding of renal cell carcinoma, but hypovascular renal cell carcinoma cannot be excluded. Hence, further MRI study is needed and renal lymphoma usually shows isointense or slight hypointense masses on T1-weighted images and definite hypointense masses on T2-weighted images as compared with the signal intensity of the renal cortex [11]. DWI, a type of MRI image, allows visualization and measurement of the random (Brownian) extracellular and intracellular motion of water molecules driven by their internal thermal energy, and it is higher signal in high cell density lesion. Apparent diffusion coefficient (ADC) is a quantitative analysis from diffuse weight imaging. Lymphoma, known as high cell density tumor due to the feature of cell proliferation, shows a bright in DWI. In this case, MRI image showed multifocal hypovascular solid tumors of the right kidneys with high DWI signal intensity, which favors lymphoma infiltration than hypovascular renal cell carcinoma.

Based on of the results of ultrasound, CT scan, MRI image, and renal pathology, the diagnosis of lymphoma was confirmed. Beyond the pathology diagnosis, ^18^F-fluorodeoxyglucose (^18^F-FDG) PET/CT is helpful to diagnose lymphoma. Previous study indicated the higher ^18^F-FDG uptake in lymphoma lesions (SUV mean 6.37 ± 2.28) than renal clear cell carcinoma (SUV mean 2.58 ± 0.62), but similar to that of renal cell carcinoma and renal collecting duct carcinoma [12]. Hence, PET/CT is a useful tool in comparing lymphoma with other renal carcinoma [12] and is regarded as the most important advance of noninvasive assessment of lymphoma [13].

## 4. Conclusion

**Figure 4:**
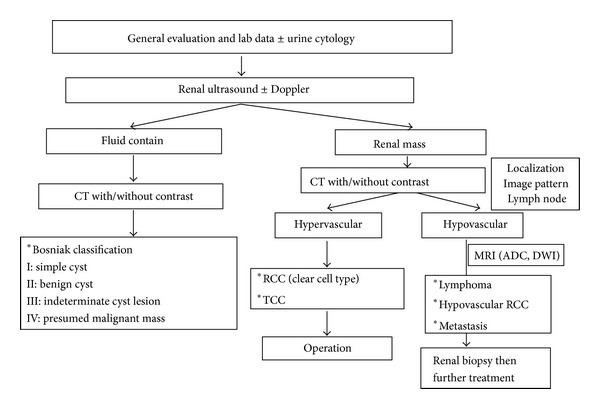
Summary of imaging survey of a renal mass. Initial imaging survey for renal mass is renal echo and if renal echo showed a renal solid mass and the next imaging survey is abdominal CT scan with/without enhancement. CT scan can detect tumor localization, imaging pattern, and lymph node involvement. If a hypovascular renal tumor is found, MRI image can help differential diagnosis the etiology. If MRI image reveals the diagnosis of lymphoma, renal biopsy is suggested.

We conduct a process to use image tool for differential diagnosis etiology of a renal mass (Figure 4). The noninvasive CT scan and MRI can help clinicians to diagnose the characteristics of renal mass and to avoid unnecessary nephrectomy. Additionally, renal biopsy is indicated for evaluation of hypovascular renal tumor. Using adequate image study and renal biopsy could help clinicians to diagnose and treat hypovascular renal tumor.
